# Long-term survival benefits of intrathecal autologous bone marrow-derived mesenchymal stem cells (Neuronata-R®: lenzumestrocel) treatment in ALS: Propensity-score-matched control, surveillance study

**DOI:** 10.3389/fnagi.2023.1148444

**Published:** 2023-04-12

**Authors:** Jae-Yong Nam, Sehwan Chun, Tae Yong Lee, Yunjeong Seo, Kwijoo Kim, Jinseok Park, Wonjae Sung, Ki-Wook Oh, Sanggon Lee, Jin-Sung Park, Juyeon Oh, Kyung Cheon Chung, Hyonggin An, Hyeon Sik Chu, Bugyeong Son, Seung Hyun Kim

**Affiliations:** ^1^Central Research Center, CORESTEMCHEMON Inc., Seoul, Republic of Korea; ^2^Department of Biology, Kyung Hee University, Seoul, Republic of Korea; ^3^College of Pharmacy, Chungbuk National University, Cheongju, Republic of Korea; ^4^Department of Neurology, College of Medicine, Hanyang University, Seoul, Republic of Korea; ^5^Department of Neurology, Chung-Ang University Gwangmyeong Hospital, Gwangmyeong, Republic of Korea; ^6^Department of Neurology, School of Medicine, Kyungpook National University, Kyungpook National University Chilgok Hospital, Daegu, Republic of Korea; ^7^College of Nursing, Dankook University, Cheonan, Republic of Korea; ^8^Department of Neurology, Bethesda Gospel Hospital, Yangsan, Republic of Korea; ^9^Department of Biostatistics, Korea University College of Medicine, Seoul, Republic of Korea; ^10^Cell Therapy Center, Hanyang University Hospital, Seoul, Republic of Korea

**Keywords:** lenzumestrocel, amyotrophic lateral sclerosis, stem cell therapy, survival analysis, mesenchymal stem cell

## Abstract

**Objective:**

Neuronata-R® (lenzumestrocel) is an autologous bone marrow-derived mesenchymal stem cell (BM-MSC) product, which was conditionally approved by the Korean Ministry of Food and Drug Safety (KMFDS, Republic of Korea) in 2013 for the treatment of amyotrophic lateral sclerosis (ALS). In the present study, we aimed to investigate the long-term survival benefits of treatment with intrathecal lenzumestrocel.

**Methods:**

A total of 157 participants who received lenzumestrocel and whose symptom duration was less than 2 years were included in the analysis (BM-MSC group). The survival data of placebo participants from the Pooled-Resource Open-Access ALS Clinical Trials (PROACT) database were used as the external control, and propensity score matching (PSM) was used to reduce confounding biases in baseline characteristics. Adverse events were recorded during the entire follow-up period after the first treatment.

**Results:**

Survival probability was significantly higher in the BM-MSC group compared to the external control group from the PROACT database (log-rank, *p* < 0.001). Multivariate Cox proportional hazard analysis showed a significantly lower hazard ratio for death in the BM-MSC group and indicated that multiple injections were more effective. Additionally, there were no serious adverse drug reactions found during the safety assessment, lasting a year after the first administration.

**Conclusion:**

The results of the present study showed that lenzumestrocel treatment had a long-term survival benefit in real-world ALS patients.

## Introduction

Amyotrophic lateral sclerosis (ALS) is a progressive neurodegenerative disease affecting both the upper and lower motor neuron systems, which eventually results in generalized weakness and, ultimately, death due to respiratory failure (Brown and Al-Chalabi, 2017; Hardiman et al., 2017).

Despite recent advances in determining the genetic and molecular mechanisms of motor neuron cell death in ALS, precise mechanisms of the selective degeneration of motor neurons and heterogeneous clinical phenotypes are not clearly understood (Taylor et al., 2016; Mejzini et al., 2019; Kim et al., 2020; Saez-Atienzar et al., 2021). Additionally, no curative therapeutic agents are currently available for the treatment of ALS. The only two Food and Drug Administration (FDA)-approved drugs are riluzole and edaravone (Miller et al., 2002; Rothstein, 2017). However, they have modest therapeutic effects (Jaiswal, 2019; Saitoh and Takahashi, 2020; Witzel et al., 2022).

Stem cell therapy has shown various potential therapeutic effects in a range of diseases along with safety in recent clinical trials (Hoang et al., 2022). In particular, the immunomodulatory abilities of mesenchymal stem cells are being investigated in many clinical trials as a treatment option for neurodegenerative diseases (Chen et al., 2018). The safety of MSC therapy has been confirmed in many studies, and some studies have shown positive results (Mazzini et al., 2010; Petrou et al., 2016; Syková et al., 2017; Aljabri et al., 2021; Tavakol-Afshari et al., 2021). There are also active clinical trials investigating the use of mesenchymal stem cell therapy for ALS.

Neuronata-R® (lenzumestrocel) is an autologous bone marrow-derived mesenchymal stem cell (BM-MSC) product, which has been conditionally approved by the Korean Ministry of Food and Drug Safety (KMFDS, December 31, 2013) as an orphan drug for concomitant therapy with riluzole for use in the treatment of patients with ALS. Phase I and II clinical trials (NCT01363401) showed clinically significant improvements in the decline of Revised ALS Functional Rating Scale (ALSFRS-R) scores up to 6 months after the administration of lenzumestrocel (Oh et al., 2015, 2018). Despite the clinical effectiveness of lenzumestrocel administration, which was determined by the decline of ALSFRS-R scores lasting at least 6 months, the lack of a long-term survival benefit in the post-hoc analysis may be associated with the limited number of injections (two doses in a single-cycle) and the gradual loss of MSC itself. Additionally, the number of participants in the phase II trial was relatively small when analyzing the survival data.

After conditional approval from the KMFDS to administer BM-MSC for the treatment of ALS, 257 participants with ALS underwent single-cycle lenzumestrocel treatment (two repeated MSC injections with 1-month interval). Among them, 34 participants with ALS received one or more booster injections at various intervals following the single-cycle treatment to achieve long-lasting benefits.

The present study aimed to provide a long-term survival analysis for lenzumestrocel treatment participants. Propensity-score-matched external control group from Pooled-Resource Open-Access ALS Clinical Trials (PROACT) were compared with those of the BM-MSC treatment group (Atassi et al., 2014).

## Materials and methods

### Lenzumestrocel surveillance study

Lenzumestrocel was developed based on the MSC properties to have the capacity to release neurotrophic factors and to show immune-inflammatory modulation in ALS, as described in previous reports (Oh et al., 2018; Nam et al., 2022). Based on the efficacy and safety data in phase I and II clinical trials (NCT01363401), lenzumestrocel was designated as an orphan drug for concomitant therapy with riluzole in patients with ALS under the Revised Rule of Orphan Drug Designation by the KMFDS (December 31, 2013; KMFDS Announcement No. 2013–262). In addition, a new drug application (NDA) for lenzumestrocel as an orphan drug was granted by the KMFDS (July 30, 2014). After NDA approval, according to the risk management plan of lenzumestrocel recommended by the KMFDS, post-marketing surveillance (PMS) was conducted as a complete enumeration survey from March 2, 2015 to January 31, 2022. This study was approved by the Hanyang University Seoul Hospital Institutional Review Board (IRB) (IRB file#: PMS2015-001).

### BM-MSC treatment cohort for propensity-score matching

**Figure 1:**
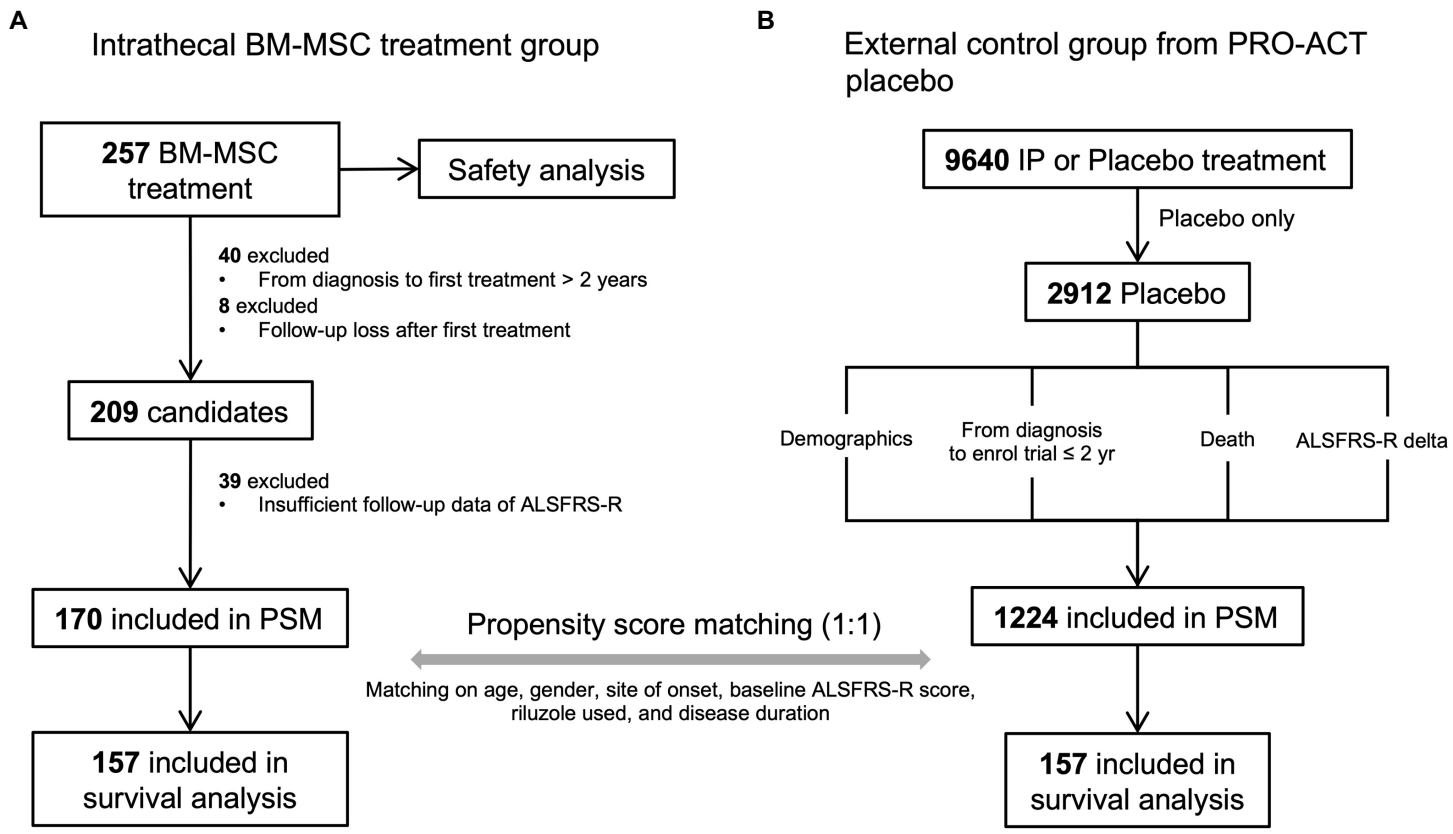
The propensity-score-matched participant selection for survival and safety analysis and data pre-processing steps. **(A)** BM-MSC treatment cohort. A total of 257 participants were administered lenzumestrocel. Safety evaluation was conducted during a one-year follow-up period after the first treatment. Survival analysis was performed on propensity score matched 157 participants. **(B)** external control group from the PROACT database. Only 2,912 placebo participants were extracted out of 9,640 participants in the treatment group data (treatement.csv). Participants who enrolled in the trial within 2 years from their diagnosis and who had a baseline ALSFRS-R score were assigned as the control group. The clinical information of each participant was extracted from each data source. A total of 157 placebo participants were used for survival analysis.

All participants of the BM-MSC treatment group were diagnosed with clinically definite or probable or clinically probable with lab-supported ALS according to the revised El Escorial criteria (Brooks et al., 2000). Clinical data (age, sex, site of onset, etc.) were collected from 257 participants treated with lenzumestrocel. To reduce the heterogeneity of the study population, participants whose symptom duration exceeded 2 years before the BM-MSC injection and who had no serial ALSFRS-R scores for the follow-up period after the first treatment were excluded from the analysis. Finally, 170 participants were included in the propensity-score matching analysis (Figure 1A). Written informed consent was obtained from all participants prior to the first administration.

### External control cohort from PROACT database for propensity-score matching

PROACT database contains de-identified records of participants from 23 clinical trials. To compare the survival probability of the BM-MSC treatment group with the placebo-allocated participants’ group, data (*n* = 2,912) were extracted from the PROACT database and used as an external control group. First, participants of the external control group whose symptom duration was less than 2 years were selected to match the BM-MSC treatment group. Then, the individuals’ death status and time were achieved from the deathdata.csv dataset. If the participant was alive until the end of the trial, the last follow-up time was determined based on the last measured time of the ALSFRS-R score from the alsfrs.csv dataset and was regarded as censored. Demographics and clinical information, such as age, sex, site of onset, and riluzole use, were obtained from other PROACT datasets. Finally, 1,224 participants were included in the propensity-score matching analysis (Figure 1B).

### Selection of BM-MSC treatment group and external control group for survival analysis using propensity score matching

Propensity score matching (PSM) is widely used in observational studies to reduce confounding biases in treated and untreated participants (Austin, 2011; Austin et al., 2021). BM-MSC-treated participants were matched to PROACT placebo participants in a ratio of 1:1 (Austin, 2010). PSM was performed using the nearest neighbor (NN) method on the propensity score, which was calculated by logistic regression, including covariates with age, sex, site of onset, baseline ALSFRS-R score, riluzole used, and disease duration. After matching, 314 participants (157 participants in each group) were included in the survival analysis.

### Estimation of initial progression speed

Due to the lack of long-term follow-up data prior to baseline for ALSFRS-R scores in both groups and inaccurate time from the onset for some subjects, accurate disease progression cannot be calculated. Therefore, the initial progression speed was calculated using the following process.

(i) Baseline ALSFRS-R is available for all subjects. (ii) Data on time from diagnosis to baseline are available for all subjects. (iii) According to the results of the PROACT study, the average time from onset to diagnosis is 12 months (Atassi et al., 2014).


$$\text{initial progression speed}=\frac{48-(\text{baseline ALSFRS}-R\;\text{score})}{\text{time from onset to baseline}\;\left(\text{months}\right)}$$

$$\text{time from onset to baseline}=\left[\text{time from the diagnosis to baseline}+12\right]\;\left(\text{months}\right)$$

### Safety assessment

Safety was evaluated based on the incidence of adverse events (AEs), adverse drug reactions (ADRs), and serious adverse events (SAEs), which were collected during the entire follow-up period from 2015 to 2022 after the first lenzumestrocel treatment. Safety analysis was conducted on the AEs that occurred one year after administration in 257 subjects who had received at least one lenzumestrocel injection. All AEs and SAEs reported within a year of the lenzumestrocel injection were considered ADR unless a causal relationship with lenzumestrocel was determined as an ‘unlikely possibility’. In addition, newly advanced neurological symptoms related to the natural progression of ALS were not regarded as AE. All AEs were coded using the Medical Dictionary for Regulatory Activities (MedDRA) version 25 and summarized according to system organ class (SOC) and preferred terms (PT; Brown et al., 1999).

### Statistical method

Differences in baseline clinical variables between the two independent groups were analyzed using a T-test (continuous) or Chi-square test (categorical) for appropriate data types. Statistical significance was set at 0.05. Survival analysis (time to death) was conducted using Kaplan–Meier curves and log-rank test using the survminer (version 0.4.9) and survival (version 3.1–8) R packages.

The BM-MSC treatment group includes participants treated with a single-cycle (repeated two injections of lenzumestrocel, *n* = 134) and a single-cycle with additional booster injections (*n* = 21, minimum three times to maximum ten times). To compare the clinical benefits of multiple treatments with lenzumestrocel, we separated the BM-MSC treatment group into a single-cycle injection group and a multiple-injection group. Two participants who were administered only once (one of two repeated injections in a single cycle) were excluded from the analysis.

The Cox proportional hazards model was used to estimate the associations between prognostic clinical variables and survival time. In the Cox model, the BM-MSC treatment group was divided into a single-cycle injection group and a multiple-injection group. We included six factors (sex, site of onset, riluzole used, baseline ALSFRS-R score, age, and treatment group) in the model to adjust for the bias of confounding factors introduced by subgrouping. Two continuous variables, age and baseline ALSFRS-R score, were dichotomized at the age of 54 years and the baseline ALSFRS-R score of 31 points. Propensity score calculations and matching were conducted using MatchIt (version 4.4.0) R package. All statistical analyses were performed using R software (version 3.6.3).

## Results

### Comparison of baseline characteristics in propensity-score-matched groups

Baseline clinical characteristics between the propensity-score-matched (PSM) BM-MSC treatment group and the external control group are summarized in Table 1. There were no statistically significant differences in baseline covariates between the matched groups. The standardized mean differences in all covariates were less than 0.1, which also indicated that the propensity score-based matching groups were well-balanced (Supplementary Figure 1).

**Table 1:** Baseline characteristics of the propensity score matched the BM-MSC treatment group and the external control group.

	BM-MSC treatment group (*n* = 157)	External control group (PROACT) (*n* = 157)	Value of *p*
Age (yr)	55.8 ± 10.7	55.7 ± 12.4	0.934
Sex (M: F)	95: 62	94: 63	1.000
Riluzole used	141 (89.8%)	141 (89.8%)	1.000
Baseline ALSFRS-R score	35.5 ± 5.90	35.7 ± 5.75	0.749
Time from diagnosis to baseline (months)	8.34 ± 5.26	8.66 ± 5.72	0.615
Site of symptom onset (Bulbar: Limb)	34: 123	31: 126	0.781

### Long-term survival analysis

**Figure 2:**
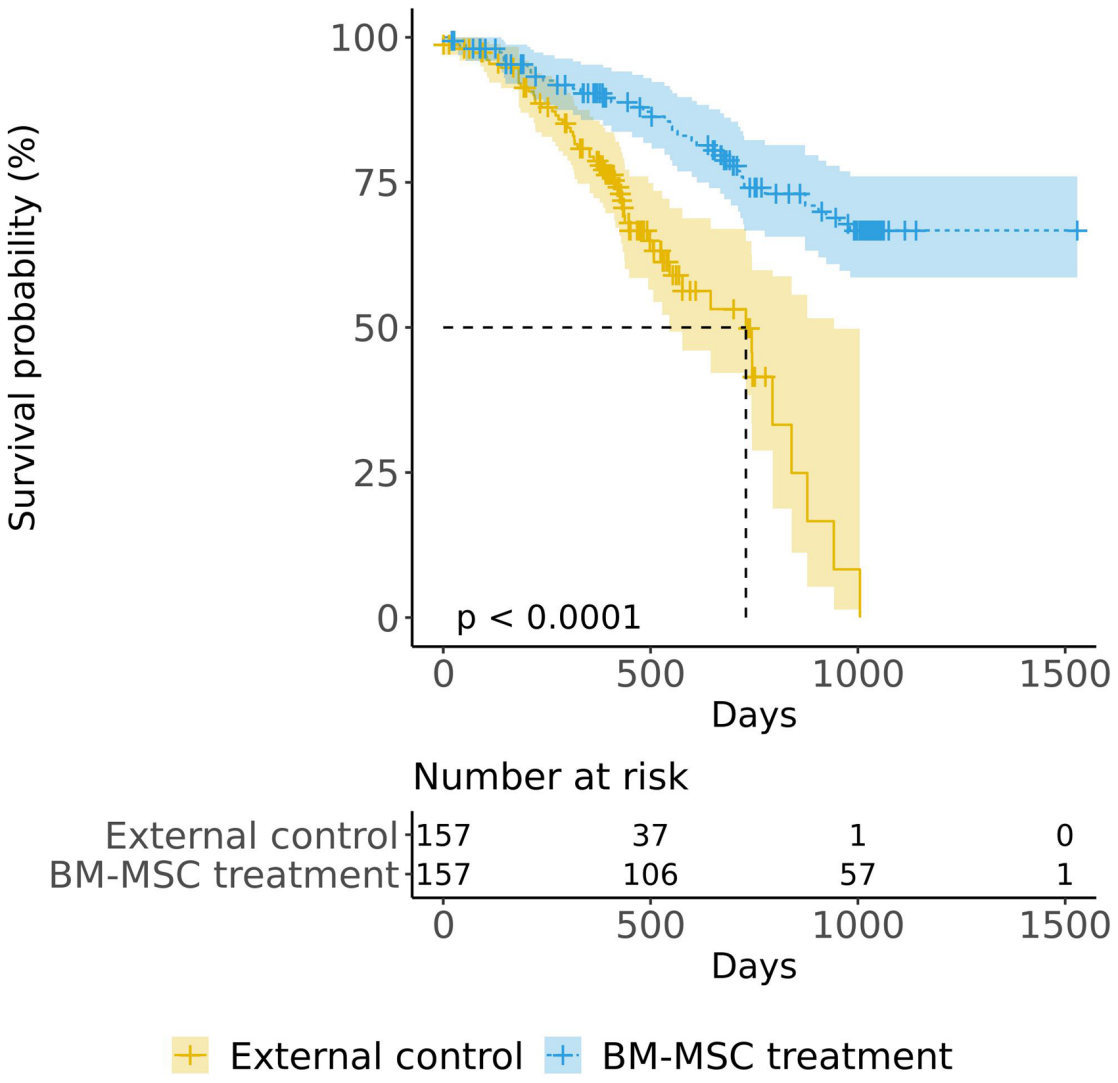
Kaplan–Meier survival curves of the BM-MSC treatment group (overall) and the external control group. The log-rank test was used to compare the survival probability between the BM-MSC treatment (blue) and the external control (yellow) groups.

In both the matched groups, we confirmed 40 deaths in the BM-MSC treatment group and 56 deaths in the external control group. The Kaplan–Meier plot of survival data shows a separation of the survival curves with significance (Figure 2). The survival probability was significantly higher in the BM-MSC treatment group compared to that in the external control group (log-rank, *p* < 0.001). The estimated median survival time of the external control group was 730 days (24 months; CI:17.95–30.97), whereas the BM-MSC treatment group’s time was not estimated as the number of death did not exceed half of the group. We also confirmed that the restricted mean survival time of the external control group was 629 days (20.68 months; SE:1.03), and the BM-MSC treatment group was 1,201 days (39.48 months; SE:1.44).

**Figure 3:**
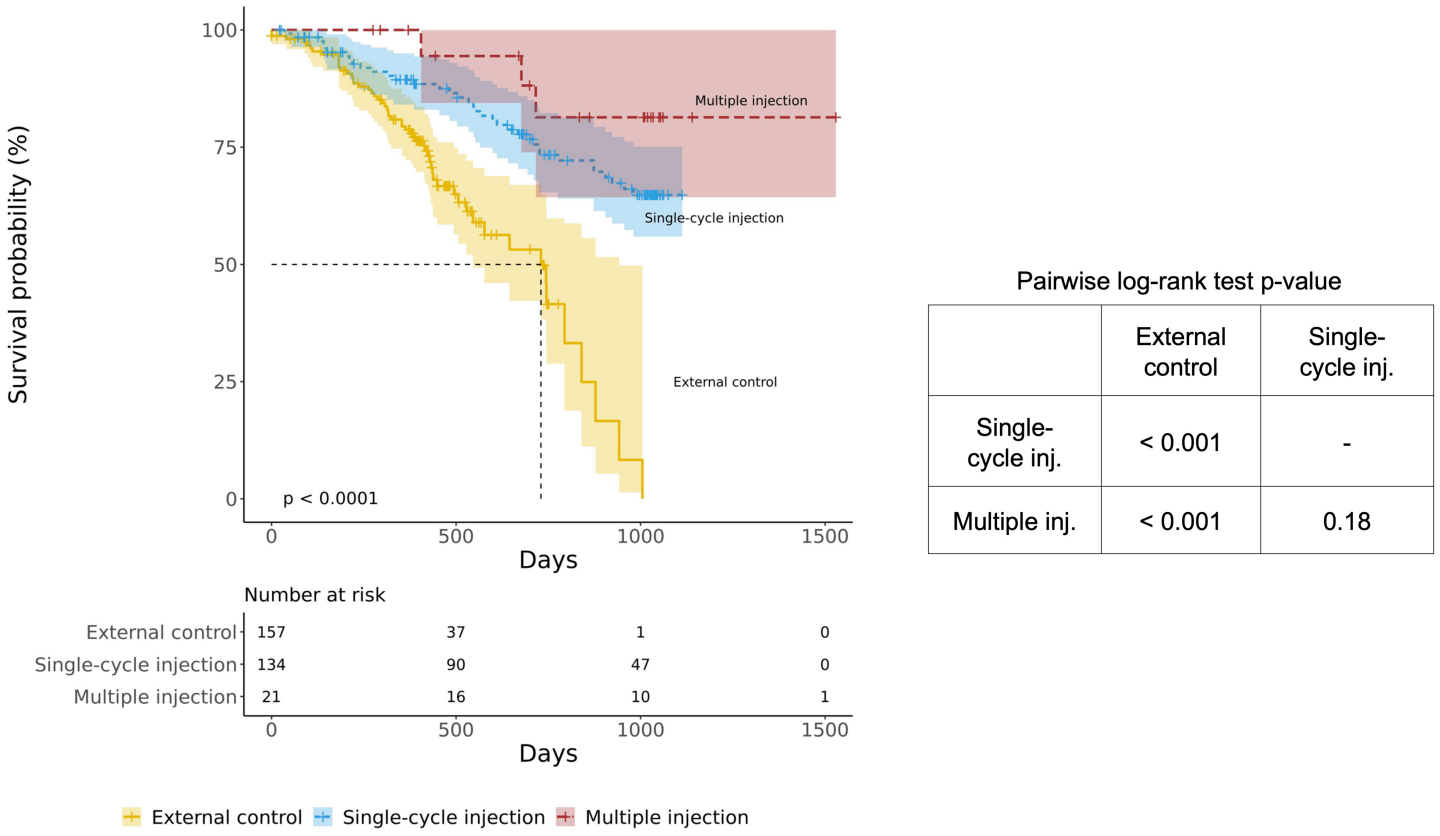
Kaplan–Meier survival curves of the BM-MSC treatment group (single-cycle injection and multiple injections) and the external control group. The BM-MSC group was divided into single-cycle injection (blue) and multiple-injection (red) groups and compared with the external control group (yellow).

The survival probabilities of both the single-cycle injection group (*p* < 0.001) and the multiple-injection group (p < 0.001) were significantly different from those of the external control group (Figure 3). It was also noted that the multiple-injection group showed a higher survival probability than the single-cycle injection group, but the difference was not statistically significant (pairwise log-rank test *p* = 0.18).

Generally, the time to events (use of tracheostomy or ventilator) is more likely to occur earlier than the time to death. The PROACT database only contains information on time to death and does not provide information on time to events. Therefore, we compared the BM-MSC group’s time-to-events information with PROACT’s time-to-death information. As a result, we confirmed that the survival probability of the BM-MSC group was higher, which was consistent with the analysis of time-to-death (Supplementary Figure 2).

### Survival analysis with initial progression speed

One of the important prognostic factors for predicting survival in ALS is the progression speed of the disease (Kimura et al., 2006; Kjældgaard et al., 2021). The disease progression speed can differ between the subjects in the two groups, which can lead to differences in survival probability. Therefore, we compared the initial progression speed between the two groups to determine whether this factor contributes to the observed difference in survival probability (see methods). The calculated initial progression speed was 0.78 ± 0.52/month for the external control group and 0.64 ± 0.32/month for the BM-MSC treatment group, indicating a difference of 0.14/month with the BM-MSC treatment group being lower (value of *p* = 0.005). The initial progression speed showed a significant difference between the BM-MSC treatment group and the external control group, which could have had an impact on the difference in survival probability. However, we added initial progression speed as a covariate to the existing PSM method and conducted survival analysis in the same manner. The results were consistent with the previous findings (Supplementary Figure 3).

Next, we compared the survival rates between the external control and BM-MSC group using only subjects with intermediate initial progression speed (between the first quartile (1Q) and the third quartile(3Q)) by limiting the range of initial progression speed (Supplementary Figure 4A). Similarly, the survival rate of the BM-MSC group was significantly higher (Supplementary Figure 4B). In conclusion, although there was a difference in initial progression speed in both groups, we confirmed that the survival probability of the BM-MSC treatment group was higher even after controlling for it through PSM or excluding rapid and slow progression.

### Hazard ratios for death in multivariate cox proportional hazards regression

**Figure 4:**
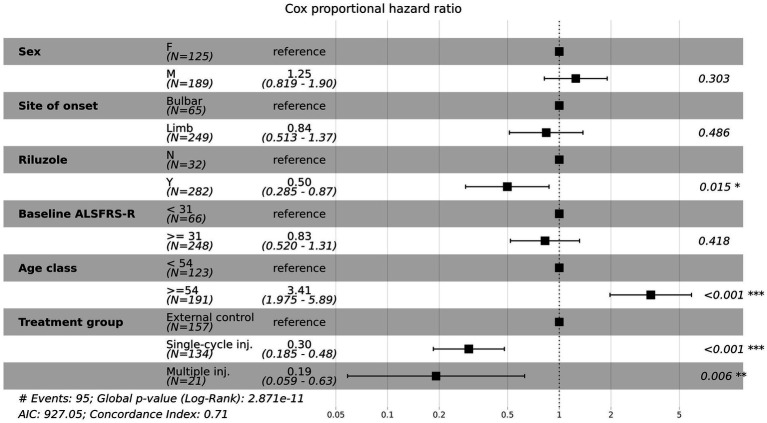
Forest plot for multivariate Cox proportional hazards model. Hazard ratios (HRs) and 95% confidence intervals (CIs) were calculated for the risk factors associated with time to death.

We conducted a Cox proportional hazards regression analysis to investigate the association between survival time and several risk factors. A forest plot of the Cox proportional hazards model is shown in Figure 4. The hazard ratios of both the single-cycle injection group (HR = 0.30, 95% CI:0.185–0.48, *p* < 0.001) and multiple-injection group (HR = 0.19, 95% CI:0.059–0.63, *p* = 0.006), which were adjusted by sex, site of onset, riluzole used, baseline ALSFRS-R score, and age, were significantly lower than the external control group (i.e., the reductions in risk of death for single-cycle injection and multiple injections were 70 and 81%, respectively). Moreover, the multiple-injection group had a lower hazard ratio than the single-cycle injection group. We found several prognostic factors associated with survival time, including riluzole use (HR = 0.50, 95% CI:0.285–0.87, *p* = 0.015) and age (HR = 3.41, 95% CI:1.975–5.89, *p* < 0.001). These results suggest that participants who received lenzumestrocel treatment have a reduced risk of death compared with the propensity-score-matched external control group. Multiple injections may have more effectiveness in patients with ALS.

### Comparison of functional assessments

We compared the change in ALSFRS-R scores from baseline to 12 months between the two groups. There was no significant difference in the change of ALSFRS-R scores between the BM-MSC group and the external control group (Supplementary Figure 5). On the other hand, when comparing the multiple-injection group and the single-cycle injection group, we observed that multiple-injection group had a lower decrease in ALSFRS-R scores (Supplementary Figure 6).

### Subgroup analysis of the differences between Korean and non-Korean subjects in the BM-MSC group

We analyzed the differences in survival probability and baseline characteristics between Korean and non-Korean subjects in the BM-MSC group. There was no significant difference in survival curves between the two groups (Supplementary Figure 7). Non-Korean subjects had a higher initial survival probability, but it decreased after crossover. At baseline, non-Korean subjects were younger (not significant) and had a 2.5-point higher ALSFRS-R score than Korean subjects (Supplementary Table 1). The initial progression speed also showed that non-Korean subjects had a slightly slow progression speed, indicating that overall, non-Korean participants had an advantage in survival. Still, the decrease in survival rate after about 1.6 years may be due to various factors, including the genetic, environmental, lifestyle, and influence of the medical system.

### Subgroup analysis of the differences between non-Korean subjects in the BM-MSC group and external control

We compared the non-Korean subjects (*n* = 25) in the BM-MSC group with an external control group. The survival probability of the BM-MSC group tended to be higher, but there was no statistically significant difference (Supplementary Figure 8).

### Long-term safety assessment

A total of 1,204 adverse events (AEs) were reported during a one-year follow-up period in 257 participants after the first administration of lenzumestrocel. Table 2 lists the AEs experienced by more than 5% of participants. The most common AEs were back pain (*n* = 85; 114 events), headache (*n* = 80; 109 events), pyrexia (*n* = 65; 91 events), pain (*n* = 45; 52 events), and pain in the extremities (*n* = 44; 53 events). The incidence of adverse drug reactions (ADRs) was 17.12% (44/257; 92 events; Table 3). The ADRs included pyrexia (*n* = 19; 21 events), headache (*n* = 19; 20 events), back pain (*n* = 12; 17 events), pain (*n* = 7; 8 events), and nausea (*n* = 6; 6 events). Most ADRs were mild and transient. However, moderate back pain, coccydynia, or pain in extremities persisting for more than 2 weeks were noted in 13 participants (5.1%; 19 events). And one case of micturition disorder (moderate and continuous) was reported. The incidence of serious adverse events (SAEs) was 26.85% (69/257; 108 events) and accounted for around 9% of AEs. Table 4 lists the SAEs experienced by more than 1% of participants. SAEs included respiratory failure (*n* = 7; 7 events), back pain (*n* = 4; 4 events), pain (*n* = 4; 4 events), coccydynia (*n* = 3; 3 events), and musculoskeletal pain (*n* = 3; 3 events). A total of 36 events were related to hospitalization, and 20 deaths occurred. All respiratory failure events were related to natural disease courses and were not considered treatment-related events. However, back pain and coccydynia may be potentially related to BM-MSC treatment due to plausible stem cell-related arachnoiditis. The AEs that were reported during the entire follow-up period are summarized in Supplementary Table 2.

**Table 2:** Summary of adverse events.

System organ class	Number of events	Number of participants	% of total participants
Preferred term			
Gastrointestinal disorders			
Constipation	32	27	10.51%
Nausea	28	24	9.34%
Dyspepsia	17	17	6.61%
Diarrhea	16	15	5.84%
General disorders and administration site conditions			
Pyrexia	91	65	25.29%
Pain	52	45	17.51%
Implant site pain	20	17	6.61%
Investigations			
Alanine aminotransferase increased	14	13	5.06%
Musculoskeletal and connective tissue disorders			
Back pain	114	85	33.07%
Pain in extremity	53	44	17.12%
Arthralgia	38	33	12.84%
Musculoskeletal pain	14	14	5.45%
Nervous system disorders			
Headache	109	80	31.13%
Dizziness	21	19	7.39%
Psychiatric disorders			
Insomnia	18	18	7.00%
Sleep disorder	13	13	5.06%
Respiratory, thoracic and mediastinal disorders			
Cough	17	14	5.45%
Productive cough	16	14	5.45%
Skin and subcutaneous tissue disorders			
Pruritus	19	17	6.61%

Only adverse events that occurred in more than 5% of all participants were listed.

**Table 3:** Summary of adverse drug reactions.

System organ class	Number of events	Number of participants	% of total participants
Preferred term			
Gastrointestinal disorders			
Nausea	6	6	2.33%
Vomiting	2	2	0.78%
General disorders and administration site conditions			
Chills	2	2	0.78%
Injection site pain	1	1	0.39%
Pain	8	7	2.72%
Pyrexia	21	19	7.39%
Injury, poisoning and procedural complications			
Upper limb fracture	1	1	0.39%
Musculoskeletal and connective tissue disorders			
Back pain	17	12	4.67%
Coccydynia	2	2	0.78%
Muscle tightness	1	1	0.39%
Musculoskeletal pain	2	2	0.78%
Myalgia	1	1	0.39%
Pain in extremity	5	5	1.95%
Nervous system disorders			
Headache	20	19	7.39%
Dizziness	2	2	0.78%
Renal and urinary disorders			
Micturition disorder	1	1	0.39%

**Table 4:** Summary of serious adverse events.

System organ class	Number of events	Number of participants	% of total participants
Preferred term			
General disorders and administration site conditions			
Pain	4	4	1.56%
Hepatobiliary disorders			
Cholecystitis acute	3	3	1.17%
Musculoskeletal and connective tissue disorders			
Back pain	4	4	1.56%
Coccydynia	3	3	1.17%
Musculoskeletal pain	3	3	1.17%
Respiratory, thoracic and mediastinal disorders			
Dyspnea	3	3	1.17%
Respiratory arrest	3	3	1.17%
Respiratory failure	7	7	2.72%

Only adverse events that occurred in more than 1% of all participants were listed.

## Discussion

Lenzumestrocel is an autologous BM-MSC isolated and expanded *ex vivo* under good manufacturing practice (GMP) conditions at CORESTEMCHEMON Inc. (Seoul, Republic of Korea). It was also conditionally approved by the Korean Ministry of Food and Drug Safety (KMFDS) for the treatment of ALS in 2013. A randomized, open-label phase II clinical trial (NCT01363401) demonstrated that single-cycle (repeated two injections with one-month interval) intrathecal administration of BM-MSCs showed a better clinical outcome (the decline of ALSFRS-R score from baseline) in the treatment group than in the control group for up to 6 months with no serious adverse drug reactions (Oh et al., 2018). In post-hoc survival analysis of the phase II clinical trial of lenzumestrocel, the estimated mean survival time was 48 (SE = 6) months in the control group and 55 (SE = 4) months in the MSC group with no significance (*p* = 0.487). The lack of long-term survival benefit may be associated with a small sample size (*n* = 64) and a relatively short observation period in the control group due to using other investigational products (Oh et al., 2018). To overcome limitations, we conducted this pilot study using a propensity-score-matched external control group.

In this study, we conducted a survival analysis of the propensity score-matched BM-MSC (*n* = 157) and external control from PROACT placebo (*n* = 157) groups to evaluate the long-term survival benefits of BM-MSC treatment in patients with ALS. The survival probability was significantly higher in the BM-MSC group than in the external control group, which is considered to indicate the long-term clinical benefit of BM-MSC treatment. In addition, the Cox proportional hazard model showed a statistically significant lower hazard ratio for both single-cycle injection and multiple injections after adjusting for prognostic covariates (e.g., sex, site of onset, riluzole used, baseline ALSFRS-R score, and age) in comparison to the external control. We also performed the same analysis on all participants before applying the PSM method and confirmed consistent results (Supplementary Figures 9, 10).

It is important to note that there were differences in baseline characteristics between the single-cycle injection and multiple-injection groups (Supplementary Table 3). The multiple-injection group had a significantly higher baseline ALSFRS-R score by approximately 4.1 points (*p* = 0.005). Additionally, there was a trend toward younger age in the multiple-injection group, but it was not significant. Furthermore, the multiple-injection group had a higher baseline ALSFRS-R score (*p* = 0.005) and a slower initial progression speed (*p* < 0.001). Therefore, it is suggested that there may be other factors contributing to the extension of survival besides multiple injections, and further investigation is necessary to identify these factors.

Due to invasive procedures of stem cell therapy, the sham-procedure control group is controversial in the early stage of the clinical trial. However, a comparison with historical control can be helpful in this situation. The age, sex, site of onset, baseline ALSFRS-R score, riluzole use, and disease duration are well-known prognostic factors of ALS. As these prognostic factors did not differ between groups in this study, trial-to-trial variations may be reduced. Therefore, despite a non-randomized study design, the two groups compared in this study seem to be appropriately balanced.

This study has the following limitations. First, the study was not designed as a two-arm randomized trial. The PROACT data is limited to patients with ALS who meet specific inclusion and exclusion criteria, which may limit the generalizability of our findings to other populations. The results provide preliminary evidence that lenzumestrocel has survival benefits. Second, while PROACT provides valuable data on ALS progression and survival, it is limited in the types of outcomes that can be measured. Other important outcomes, such as quality of life and time to events (ventilator or tracheostomy), were not included in the data. Therefore, our findings should be interpreted within the context of the limitations of the PROACT dataset. Third, the Korean healthcare system may have a potential influence. Even though 16% (25/157) of participants were non-Korean, critical treatments in the course of ALS, including riluzole prescription, percutaneous endoscopic gastrostomy (PEG) tube insertion, non-invasive ventilator rental, and tracheostomy care, are covered by the Korean National Health Insurance Service, lowering the patient’s out-of-pocket expense to 10% of the overall medical cost (Kim et al., 2021). In addition, considering the cost of lenzumestrocel, the socioeconomic status of the participants is considered to be above-average and, thereby, may contribute to their survival. Fourth, most participants were followed up in a single center, one of the multidisciplinary ALS clinics in a tertiary hospital in Seoul. It is acknowledged that multidisciplinary care can increase the survival probability (Hardiman et al., 2017), and Seoul and the metropolitan area are recognized to offer superior accessibility and levels of medical service (Jun et al., 2019), which can also contribute to the survival probability. Fifth, the subjects in the multiple-injection group had various injection intervals after single-cycle administration. The analysis of cytokines in CSF of patients with different additional injection periods showed that patients with additional injections at 3–4 months had more benefits in ALSFRS-R score and cytokine levels compared to those with injection intervals of 5–12 months (Supplementary Figures 11–13). The potential difference in clinical benefits depending on the interval of additional injections was not addressed in this study. Sixth, more detailed safety issues, including long-term persistent pain after stem cell therapy, should be more systemically analyzed in larger-scale clinical trials. To overcome these limitations, a phase III trial was required to confirm the long-term efficacy of lenzumestrocel.

Phase III ALSUMMIT clinical trial protocol (NCT04745299) was approved by the U.S. FDA and KMFDS. ALSUMMIT is a randomized, multicenter, double-blind, parallel-group, sham procedure-controlled phase III trial to evaluate the long-term efficacy and safety of repeated BM-MSCs in the treatment of ALS (56-week main study with five BM-MSC injections followed by 24-month observational study) (Nam et al., 2022). The participant recruitment progress nearly reached 90% while preparing this manuscript.

In summary, our analyses revealed a statistically significant long-term survival extension of BM-MSC treatment and suggested that multiple additional booster injections may have more survival benefits in patients with ALS.

## Data availability statement

The original contributions presented in the study are included in the article/Supplementary material, further inquiries can be directed to the corresponding author.

## Ethics statement

The studies involving human participants were reviewed and approved by the Hanyang University Seoul Hospital Institutional Review Board (IRB file#: PMS2015-001). The participants provided their written informed consent to participate in this study.

## Author contributions

J-YN, TL, and SK conceptualized and designed the study. YS, KK, and BS collected the clinical data. J-YN and SC analyzed the data. J-YN, SC, and SK wrote the manuscript. J-YN, JP, WS, J-SP, JO, BS, K-WO, SL, HC, KC, and SK reviewed the manuscript. J-YN, BS, and SK revised the final version of the manuscript. HA conducted statistical analysis and review. All authors have carefully reviewed and agreed to the submitted version of the manuscript.

## Conflict of interest

J-YN, SC, TL, YS, and KK are employees of CORESTEMCHEMON Inc.

The remaining authors declare that the research was conducted in the absence of any commercial or financial relationships that could be construed as a potential conflict of interest.

## Publisher’s note

All claims expressed in this article are solely those of the authors and do not necessarily represent those of their affiliated organizations, or those of the publisher, the editors and the reviewers. Any product that may be evaluated in this article, or claim that may be made by its manufacturer, is not guaranteed or endorsed by the publisher.
