# Supplementary material for: Long-term survival benefits of intrathecal autologous bone marrow-derived mesenchymal stem cells (Neuronata-R®: lenzumestrocel) treatment in ALS: Propensity-score-matched control, surveillance study
Source: Front Aging Neurosci. 2023 Apr 12;15:1148444. doi: 10.3389/fnagi.2023.1148444 (PMC10130504; doi:10.3389/fnagi.2023.1148444)
Supplement: Supplementary file 1 [file Data_Sheet_2.docx]

Supplementary Material

Long-term Survival Benefits of Intrathecal Autologous Bone Marrow-derived Mesenchymal Stem Cells (Neuronata-R®: lenzumestrocel) Treatment in ALS: Propensity-score Matched Control, Surveillance Study

Jae-Yong Nam^1^, Sehwan Chun^1,2^, Tae Yong Lee^1,3^, Yunjeong Seo^1^, Kwijoo Kim^1^, Jinseok Park^4^, Wonjae Sung^4^, Ki-Wook Oh^4^, Sanggon Lee^4,5^, Jin-Sung Park^6^, Juyeon Oh^7^, Kyung Cheon Chung^8^, Hyonggin An^9^, Hyeon Sik Chu^10^, Bugyeong Son^10^, Seung Hyun Kim^4,10*^

*** Correspondence:** Prof. Seung Hyun Kim, MD, PhD: kimsh1@hanyang.ac.kr

# Supplementary Figures


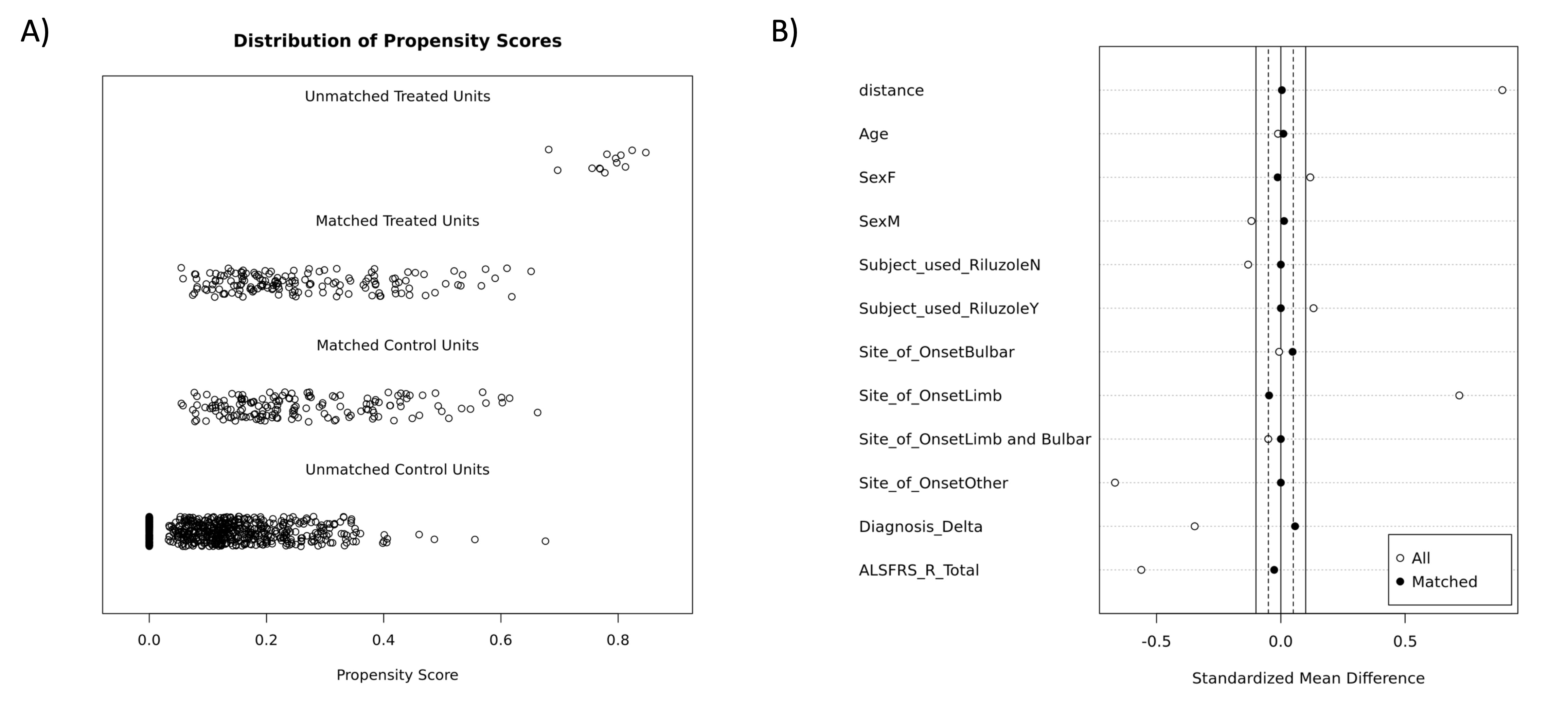


Supplementary Figure 1. Distribution of propensity scores and standardized mean differences of covariates between groups before and after propensity score matching. (A) Distribution of propensity scores in matched and unmatched participants. A total of 157 participants per group was assigned. (B) The standardized mean differences of clinical covariates in before and after propensity score matching (PSM). After matching, all clinical covariates were well-balanced.


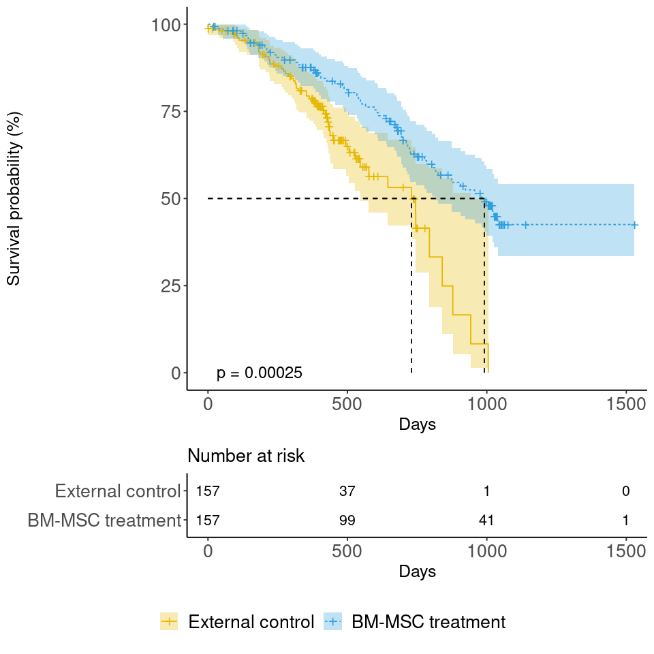


Supplementary Figure 2. Kaplan-Meier survival curves of time to events (BM-MSC) and time to deaths (external control)


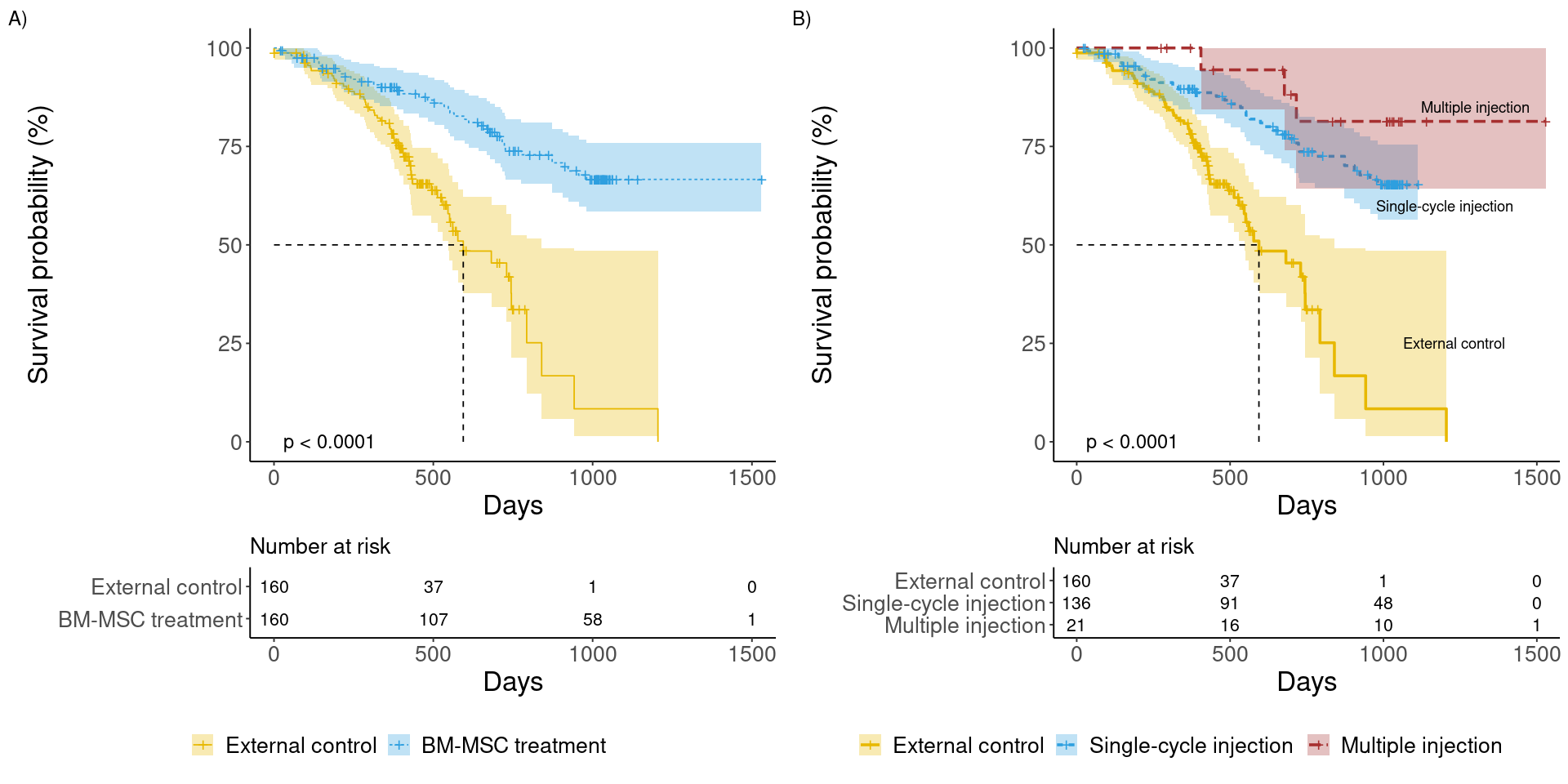


Supplementary Figure 3. Kaplan-Meier survival curves of BM-MSC and control group after adding initial progression speed as covariate to PSM. (A) Kaplan-Meier survival curves of BM-MSC and control group. (B) Kaplan-Meier survival curves of single-dose, multi-dose, and control groups.


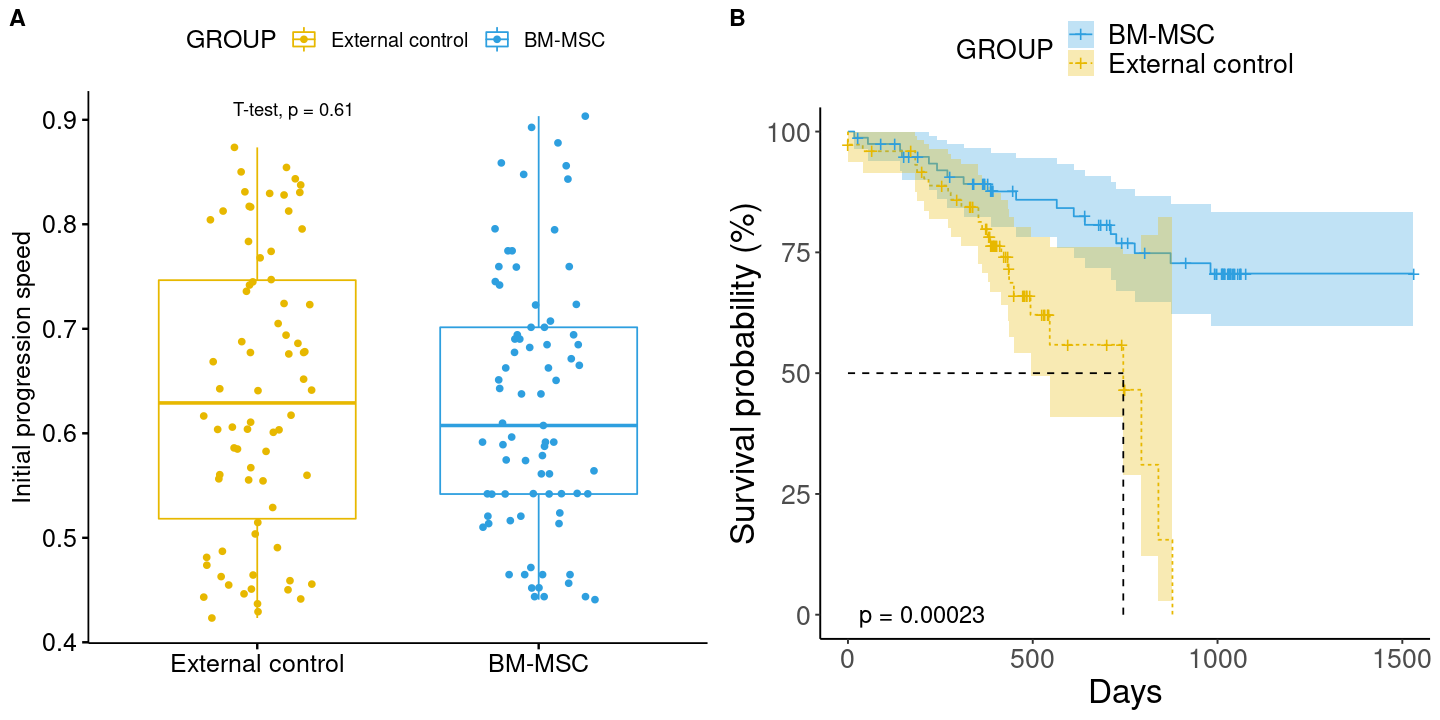


Supplementary Figure 4. Survival analysis was performed by selecting only patients with intermediate initial progression speed. (A) Boxplot of subjects with intermediate progression speed selected from two groups. (B) Kaplan-Meier survival curves of subjects with intermediate speed in both groups.


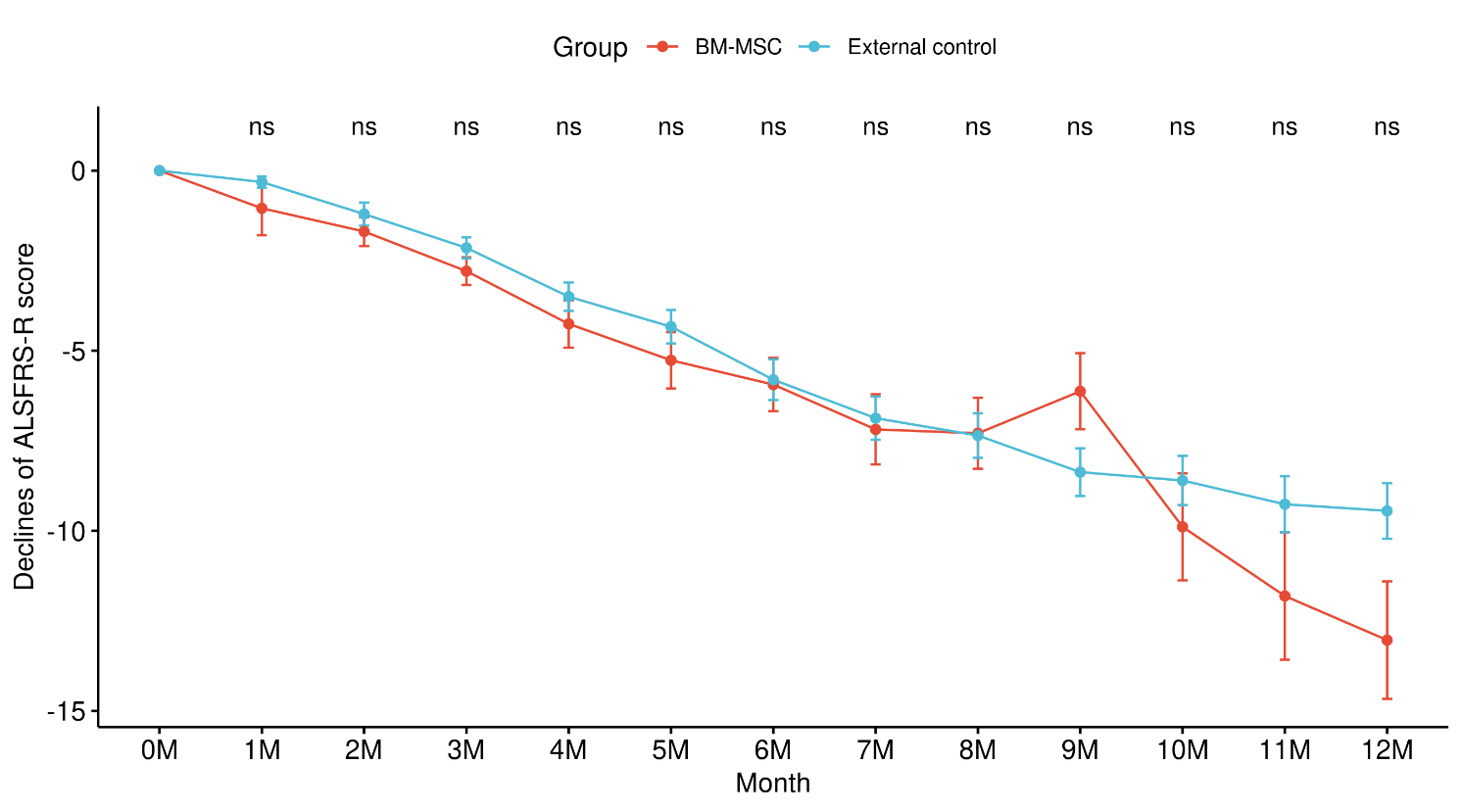


Supplementary Figure 5. Declines of ALSFRS-R score from baseline to 12 Months in BM-MSC and control. (ns: p>0.05)


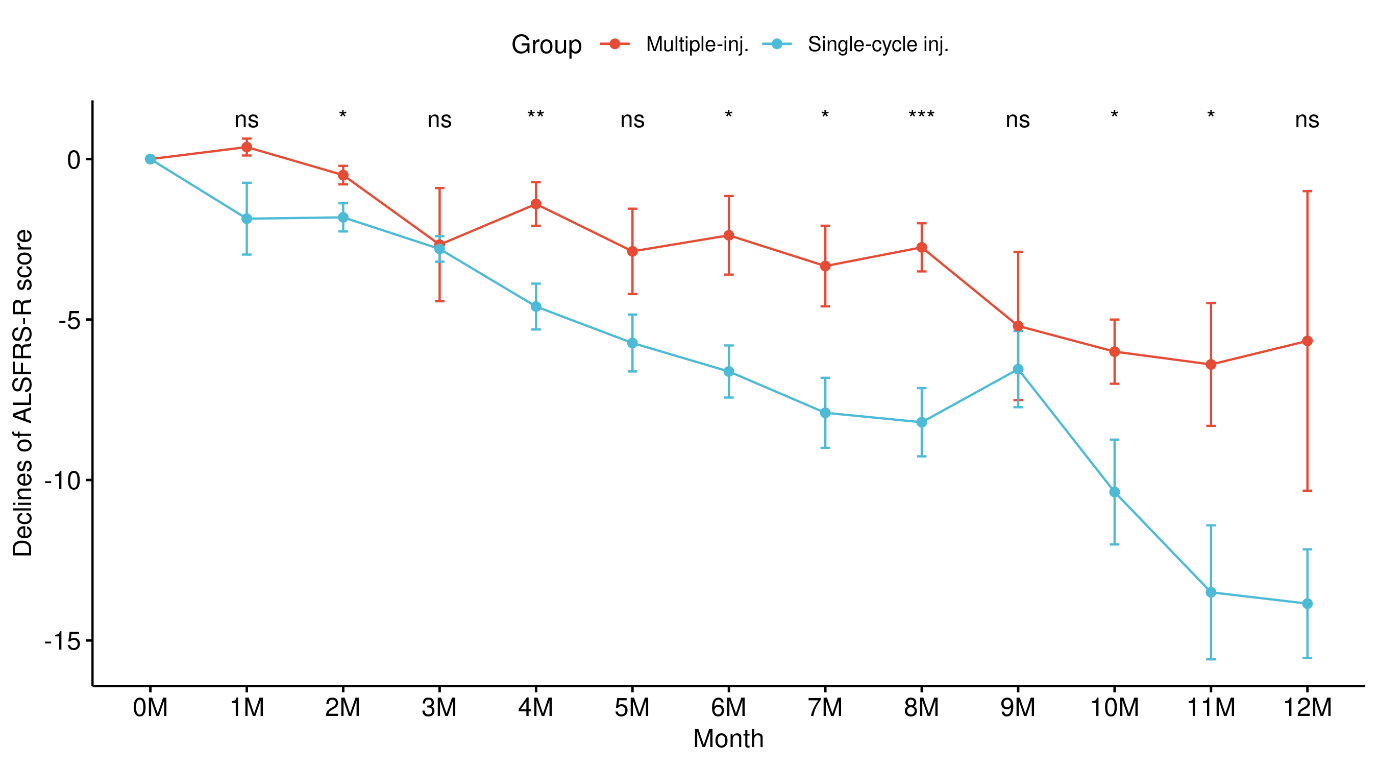


Supplementary Figure 6. Declines of ALSFRS-R scores from baseline to 12 Months in multiple-dose and single-dose in BM-MSC group. (ns: p>0.05, *: p≤0.05, **: p≤0.01, ***: p≤0.001)


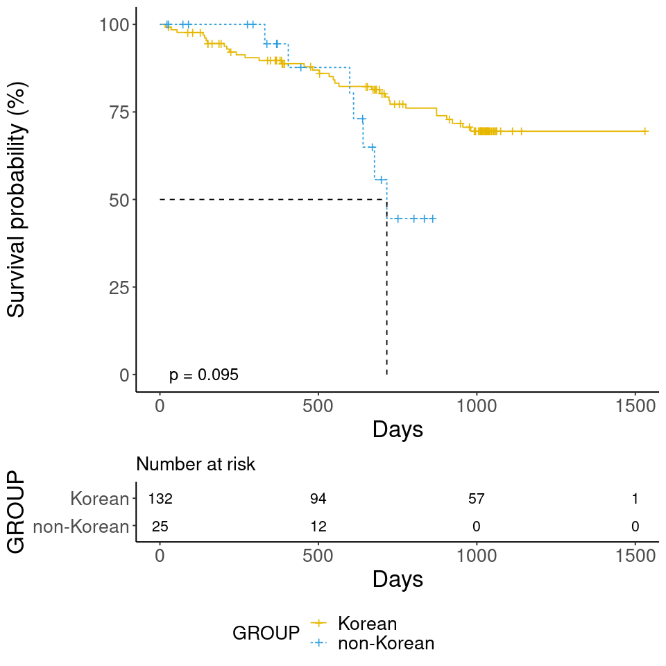


Supplementary Figure 7. Kaplan-Meier survival curves of Korean and non-Korean in the BM-MSC group


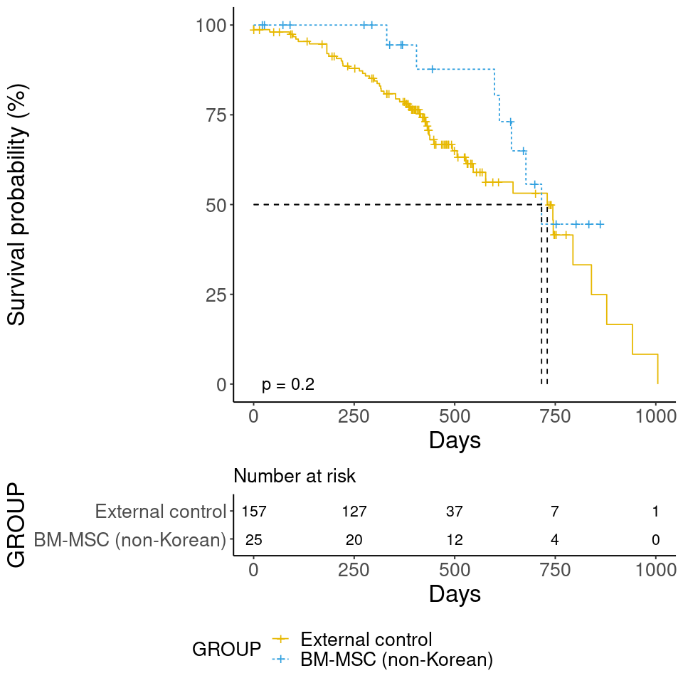


Supplementary Figure 8. Kaplan-Meier curves of non-Korean (BM-MSC) and external control.


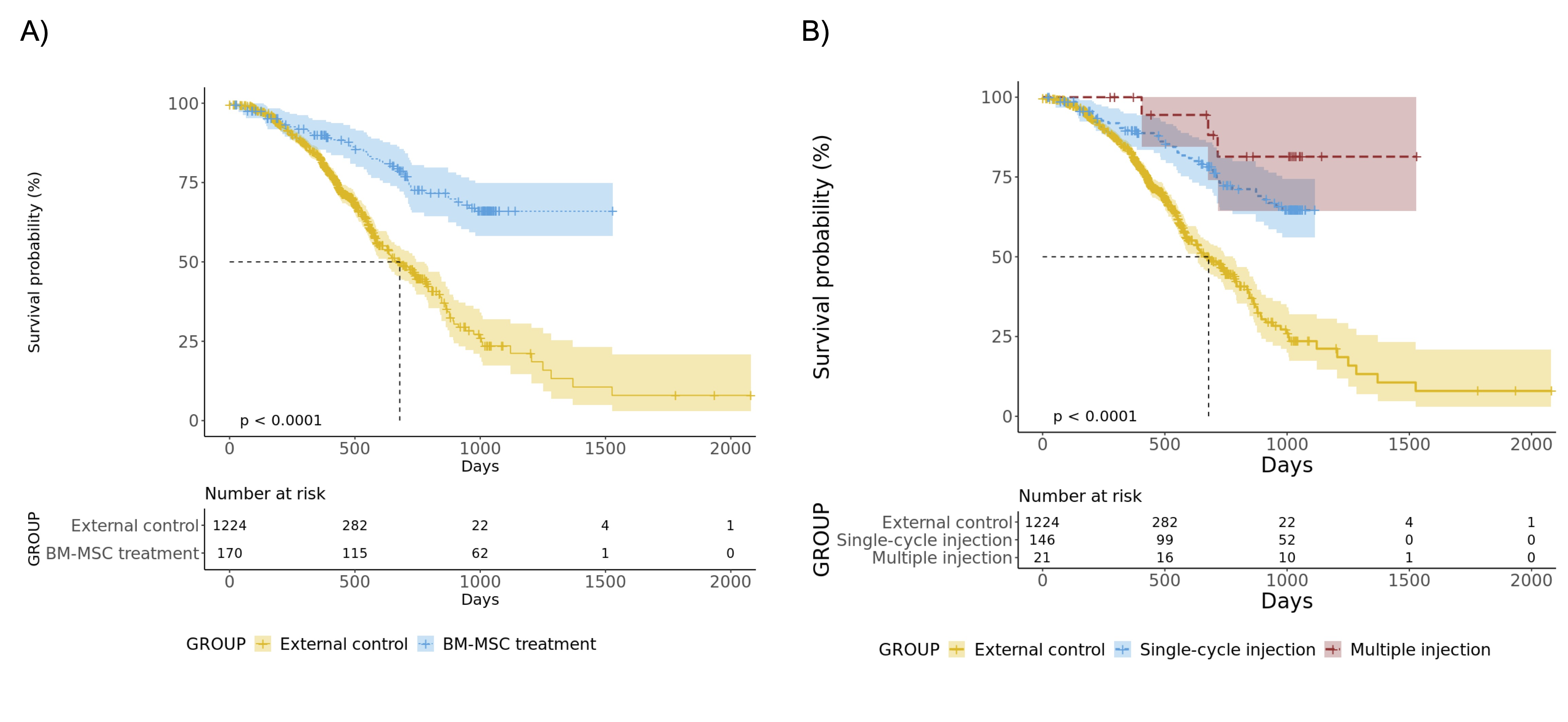


Supplementary Figure 9. Kaplan-Meier survival curves of the BM-MSC treatment group and the external control group in all participants before PSM. (A) Survival curves of the BM-MSC treatment group (overall) and the external control group. (B) Survival curves of the single-cycle injection group, multiple-injection group, and the external control group.


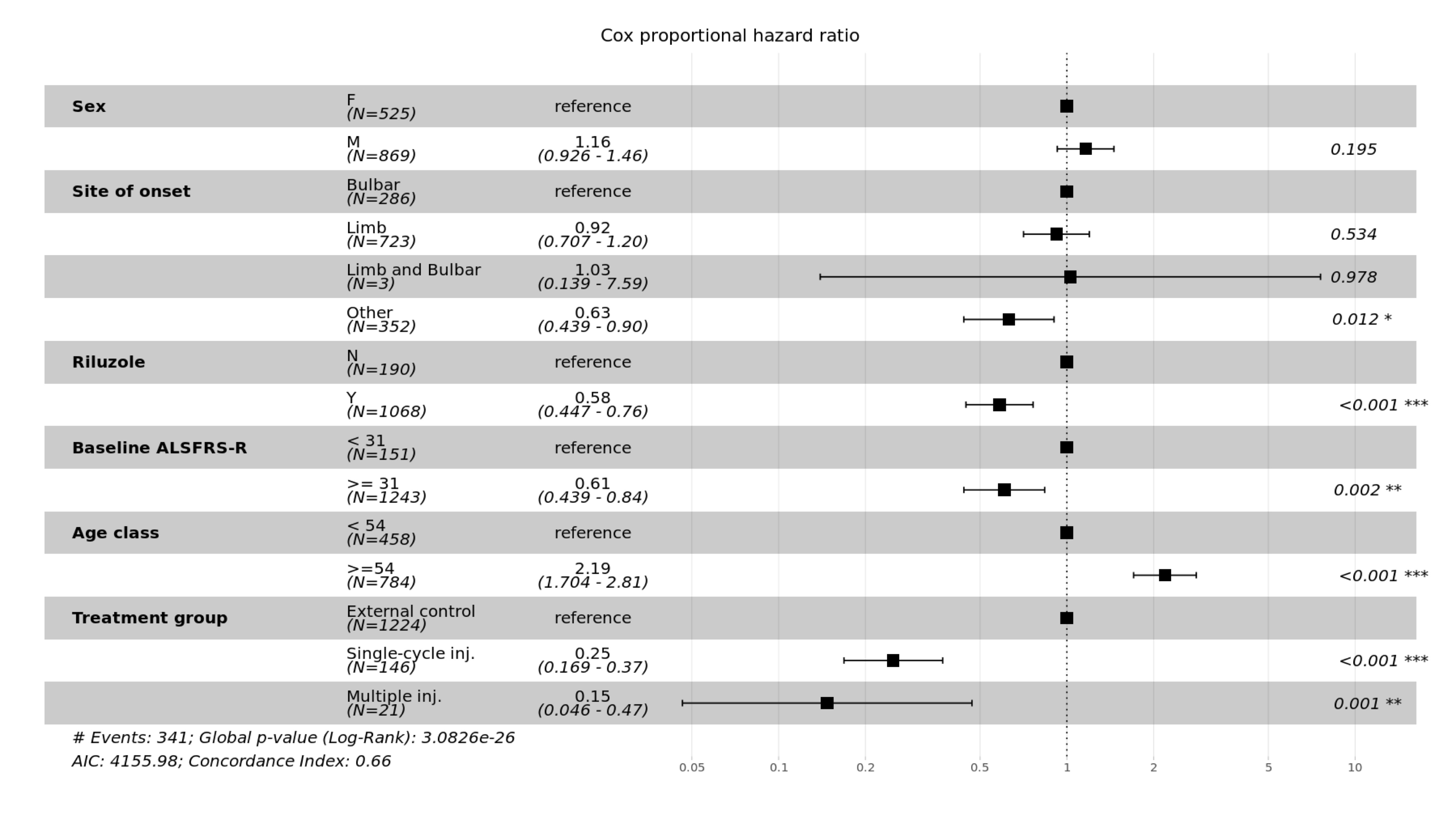


Supplementary Figure 10. Forest plot for multivariate Cox proportional hazards model in all participants before PSM.


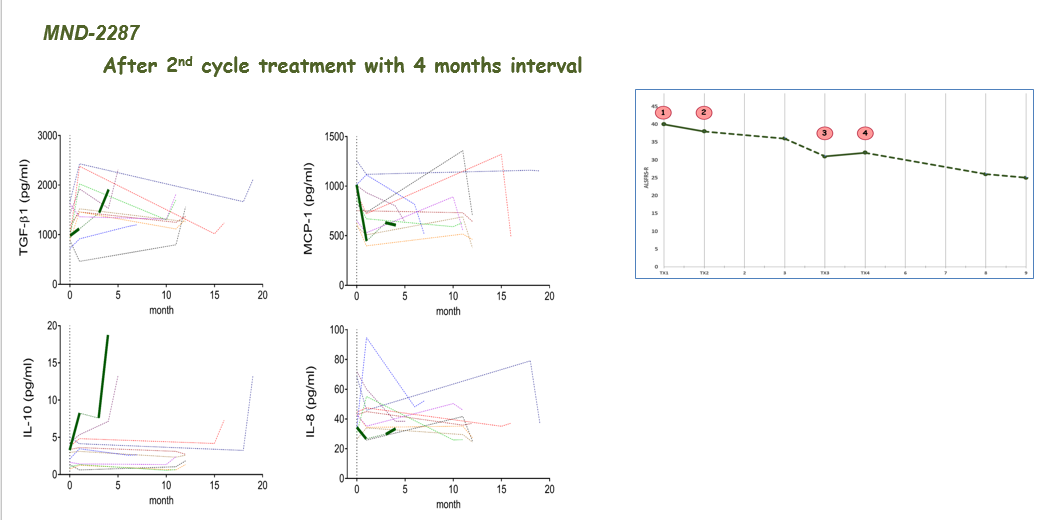


Supplementary Figure 11. The changes in cytokine levels and ALSFRS-R scores of patients who received additional injections at 4 months after single-cycle administration.


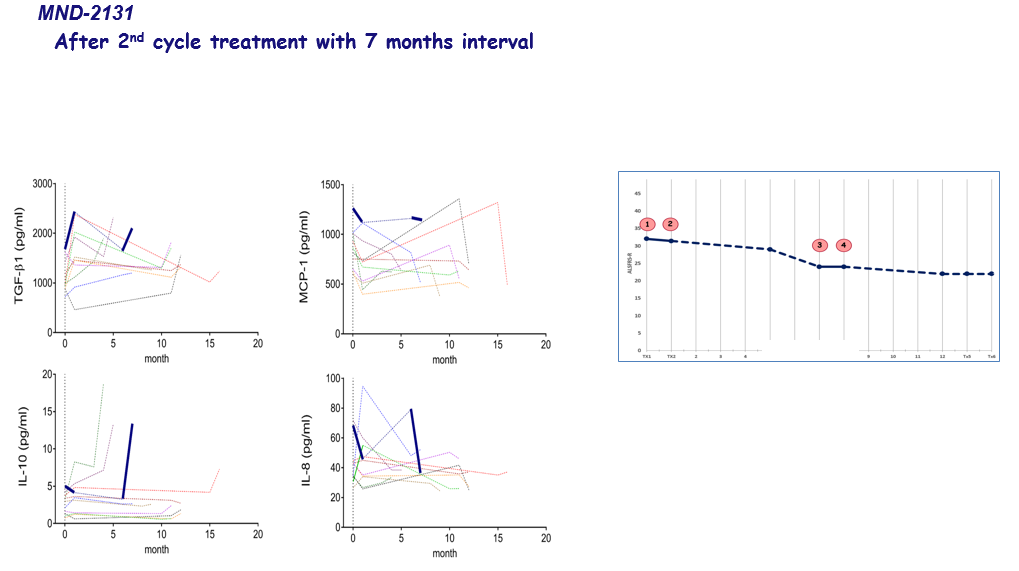


Supplementary Figure 12. The changes in cytokine levels and ALSFRS-R scores of patients who received additional injections at 7 months after single-cycle administration.


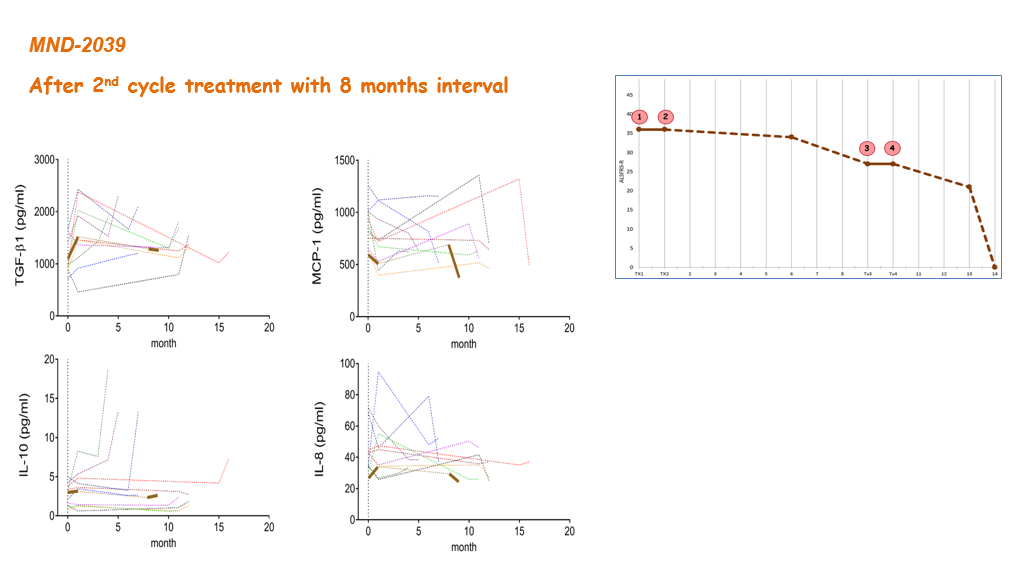


Supplementary Figure 13. The changes in cytokine levels and ALSFRS-R scores of patients who received additional injections at 8 months after single-cycle administration.

# Supplementary Table

Supplementary Table 1. Baseline characteristics of Korean and non-Korean in the BM-MSC group

|  | Korean (n=132) | non-Korean (n=25) | p-value |
| --- | --- | --- | --- |
| Age (yr) | 56.6±10.1 | 51.4±12.7 | 0.065 |
| Sex (M : F) | 77:55 (58%:42%) | 18:7 (72%:28%) | 0.290 |
| Riluzole used (%) | 118 (89%) | 23 (92%) | 1.000 |
| Baseline ALSFRS-R score | 35.1±5.97 | 37.6±5.11 | 0.031 |
| Time from diagnosis to baseline (months) | 7.99±4.90 | 10.24±6.67 | 0.119 |
| Site of symptom onset (Bulbar: Limb) | 30:102 (23%:77%) | 4:21 (16%:84%) | 0.628 |
| Initial progression speed (ALSFRS-R slope change/month) | 0.67 (0.33) | 0.50 (0.28) | 0.011 |

Supplementary Table 2. Summary of AEs during the entire follow-up period

| Adverse Events | Frequency | N | % |
| --- | --- | --- | --- |
| Blood and lymphatic system disorders | **11** | **10** | **3.89%** |
| Anaemia | 4 | 4 | 1.56% |
| Leukocytosis | 1 | 1 | 0.39% |
| Lymphadenopathy | 1 | 1 | 0.39% |
| Neutropenia | 2 | 2 | 0.78% |
| Polycythaemia | 1 | 1 | 0.39% |
| Splenic cyst | 1 | 1 | 0.39% |
| Thrombocytopenia | 1 | 1 | 0.39% |
| Cardiac disorders | **27** | **22** | **8.56%** |
| Acute myocardial infarction | 2 | 2 | 0.78% |
| Arrhythmia | 2 | 2 | 0.78% |
| Cardiac arrest | 5 | 5 | 1.95% |
| Cardiac failure | 1 | 1 | 0.39% |
| Cardiomegaly | 1 | 1 | 0.39% |
| Coronary artery disease | 2 | 2 | 0.78% |
| Palpitations | 2 | 2 | 0.78% |
| Pericardial effusion | 1 | 1 | 0.39% |
| Sinus bradycardia | 1 | 1 | 0.39% |
| Sinus tachycardia | 2 | 2 | 0.78% |
| Stress cardiomyopathy | 2 | 2 | 0.78% |
| Tachycardia | 6 | 6 | 2.33% |
| Ear and labyrinth disorders | **12** | **10** | **3.89%** |
| Cerumen impaction | 1 | 1 | 0.39% |
| Ear disorder | 1 | 1 | 0.39% |
| Ear pain | 2 | 2 | 0.78% |
| Ear swelling | 1 | 1 | 0.39% |
| Hypoacusis | 2 | 2 | 0.78% |
| Otolithiasis | 1 | 1 | 0.39% |
| Otorrhoea | 1 | 1 | 0.39% |
| Tinnitus | 1 | 1 | 0.39% |
| Vertigo positional | 2 | 1 | 0.39% |
| Endocrine disorders | **2** | **2** | **0.78%** |
| Hyperthyroidism | 1 | 1 | 0.39% |
| Thyroid mass | 1 | 1 | 0.39% |
| Eye disorders | **23** | **17** | **6.61%** |
| Abnormal sensation in eye | 1 | 1 | 0.39% |
| Cataract | 1 | 1 | 0.39% |
| Conjunctival deposit | 1 | 1 | 0.39% |
| Conjunctival oedema | 1 | 1 | 0.39% |
| Conjunctivitis allergic | 1 | 1 | 0.39% |
| Corneal erosion | 1 | 1 | 0.39% |
| Dry eye | 2 | 2 | 0.78% |
| Eye discharge | 1 | 1 | 0.39% |
| Eye inflammation | 1 | 1 | 0.39% |
| Eye pain | 5 | 5 | 1.95% |
| Eye pruritus | 1 | 1 | 0.39% |
| Foreign body sensation in eyes | 1 | 1 | 0.39% |
| Glare | 1 | 1 | 0.39% |
| Ocular discomfort | 2 | 2 | 0.78% |
| Pterygium | 1 | 1 | 0.39% |
| Saccadic eye movement | 1 | 1 | 0.39% |
| Vision blurred | 1 | 1 | 0.39% |
| Gastrointestinal disorders | **244** | **120** | **46.69%** |
| Abdominal discomfort | 4 | 4 | 1.56% |
| Abdominal distension | 3 | 3 | 1.17% |
| Abdominal pain | 16 | 12 | 4.67% |
| Abdominal pain upper | 6 | 5 | 1.95% |
| Aphthous ulcer | 1 | 1 | 0.39% |
| Chronic gastritis | 2 | 2 | 0.78% |
| Constipation | 55 | 47 | 18.29% |
| Dental caries | 1 | 1 | 0.39% |
| Diarrhoea | 27 | 25 | 9.73% |
| Dry mouth | 7 | 7 | 2.72% |
| Duodenitis | 1 | 1 | 0.39% |
| Dyschezia | 3 | 3 | 1.17% |
| Dyspepsia | 28 | 25 | 9.73% |
| Enteritis | 2 | 2 | 0.78% |
| Gastric ulcer | 1 | 1 | 0.39% |
| Gastritis | 1 | 1 | 0.39% |
| Gastritis erosive | 1 | 1 | 0.39% |
| Gastrooesophageal reflux disease | 3 | 3 | 1.17% |
| Gingival bleeding | 2 | 2 | 0.78% |
| Gingival pain | 4 | 4 | 1.56% |
| Glossodynia | 1 | 1 | 0.39% |
| Haematochezia | 1 | 1 | 0.39% |
| Haemorrhoids | 1 | 1 | 0.39% |
| Ileus | 2 | 2 | 0.78% |
| Ileus paralytic | 2 | 2 | 0.78% |
| Intestinal haemorrhage | 1 | 1 | 0.39% |
| Large intestine polyp | 1 | 1 | 0.39% |
| Melaena | 4 | 4 | 1.56% |
| Nausea | 39 | 32 | 12.45% |
| Pancreatitis | 3 | 3 | 1.17% |
| Pneumoperitoneum | 1 | 1 | 0.39% |
| Rectal tenesmus | 1 | 1 | 0.39% |
| Stomatitis | 3 | 3 | 1.17% |
| Tongue ulceration | 1 | 1 | 0.39% |
| Toothache | 4 | 4 | 1.56% |
| Vomiting | 11 | 11 | 4.28% |
| General disorders and administration site conditions | **287** | **147** | **57.20%** |
| Asthenia | 3 | 3 | 1.17% |
| Catheter site inflammation | 1 | 1 | 0.39% |
| Chest discomfort | 4 | 4 | 1.56% |
| Chest pain | 6 | 6 | 2.33% |
| Chills | 8 | 8 | 3.11% |
| Condition aggravated | 1 | 1 | 0.39% |
| Death | 17 | 17 | 6.61% |
| Face oedema | 1 | 1 | 0.39% |
| Fatigue | 1 | 1 | 0.39% |
| Feeling hot | 1 | 1 | 0.39% |
| Generalised oedema | 1 | 1 | 0.39% |
| Implant site pain | 33 | 29 | 11.28% |
| Implant site pruritus | 2 | 2 | 0.78% |
| Injection site pain | 4 | 4 | 1.56% |
| Injection site rash | 1 | 1 | 0.39% |
| Oedema peripheral | 12 | 9 | 3.50% |
| Pain | 72 | 59 | 22.96% |
| Penile oedema | 1 | 1 | 0.39% |
| Peripheral swelling | 3 | 3 | 1.17% |
| Puncture site haemorrhage | 1 | 1 | 0.39% |
| Pyrexia | 113 | 72 | 28.02% |
| Ulcer | 1 | 1 | 0.39% |
| Hepatobiliary disorders | **10** | **9** | **3.50%** |
| Cholecystitis | 1 | 1 | 0.39% |
| Cholecystitis acute | 3 | 3 | 1.17% |
| Cholelithiasis | 3 | 3 | 1.17% |
| Hepatitis toxic | 2 | 1 | 0.39% |
| Non-alcoholic steatohepatitis | 1 | 1 | 0.39% |
| Immune system disorders | **2** | **2** | **0.78%** |
| Anti-neutrophil cytoplasmic antibody positive vasculitis | 1 | 1 | 0.39% |
| Contrast media allergy | 1 | 1 | 0.39% |
| Infections and infestations | **97** | **56** | **21.79%** |
| Acarodermatitis | 2 | 2 | 0.78% |
| Bacterial vaginosis | 1 | 1 | 0.39% |
| Bacteriuria | 1 | 1 | 0.39% |
| Cellulitis | 3 | 3 | 1.17% |
| Chronic sinusitis | 1 | 1 | 0.39% |
| Conjunctivitis | 1 | 1 | 0.39% |
| Cystitis | 4 | 4 | 1.56% |
| Demodicidosis | 1 | 1 | 0.39% |
| Empyema | 1 | 1 | 0.39% |
| Fungal infection | 1 | 1 | 0.39% |
| Fungal skin infection | 1 | 1 | 0.39% |
| Herpes simplex | 2 | 2 | 0.78% |
| Herpes zoster | 6 | 6 | 2.33% |
| Hordeolum | 1 | 1 | 0.39% |
| Infection | 1 | 1 | 0.39% |
| Laryngitis | 1 | 1 | 0.39% |
| Latent tuberculosis | 1 | 1 | 0.39% |
| Onychomycosis | 5 | 5 | 1.95% |
| Oral candidiasis | 1 | 1 | 0.39% |
| Otitis externa | 1 | 1 | 0.39% |
| Otitis media | 1 | 1 | 0.39% |
| Otitis media acute | 1 | 1 | 0.39% |
| Paronychia | 1 | 1 | 0.39% |
| Periodontitis | 2 | 2 | 0.78% |
| Pharyngitis | 2 | 2 | 0.78% |
| Pneumonia | 5 | 5 | 1.95% |
| Pseudomembranous colitis | 1 | 1 | 0.39% |
| Pyelitis | 1 | 1 | 0.39% |
| Pyelonephritis | 1 | 1 | 0.39% |
| Pyelonephritis acute | 2 | 2 | 0.78% |
| Rhinitis | 2 | 2 | 0.78% |
| Scrotal abscess | 1 | 1 | 0.39% |
| Sepsis | 1 | 1 | 0.39% |
| Septic pulmonary embolism | 1 | 1 | 0.39% |
| Septic shock | 7 | 4 | 1.56% |
| Streptococcal infection | 1 | 1 | 0.39% |
| Tinea cruris | 2 | 2 | 0.78% |
| Tinea manuum | 1 | 1 | 0.39% |
| Tinea pedis | 7 | 7 | 2.72% |
| Tonsillitis | 1 | 1 | 0.39% |
| Tracheostomy infection | 1 | 1 | 0.39% |
| Tuberculosis | 1 | 1 | 0.39% |
| Upper respiratory tract infection | 5 | 4 | 1.56% |
| Urinary tract infection | 11 | 10 | 3.89% |
| Urosepsis | 1 | 1 | 0.39% |
| Varicella | 1 | 1 | 0.39% |
| Injury, poisoning and procedural complications | **53** | **41** | **15.95%** |
| Abdominal wall wound | 1 | 1 | 0.39% |
| Ankle fracture | 1 | 1 | 0.39% |
| Clavicle fracture | 3 | 3 | 1.17% |
| Contusion | 2 | 2 | 0.78% |
| Ear canal abrasion | 1 | 1 | 0.39% |
| Fall | 5 | 4 | 1.56% |
| Femur fracture | 1 | 1 | 0.39% |
| Fibula fracture | 1 | 1 | 0.39% |
| Foot fracture | 2 | 2 | 0.78% |
| Head injury | 2 | 2 | 0.78% |
| Humerus fracture | 1 | 1 | 0.39% |
| Implant site pain | 2 | 2 | 0.78% |
| Joint dislocation | 1 | 1 | 0.39% |
| Ligament sprain | 1 | 1 | 0.39% |
| Limb injury | 1 | 1 | 0.39% |
| Lower limb fracture | 1 | 1 | 0.39% |
| Lumbar vertebral fracture | 1 | 1 | 0.39% |
| Post procedural haemorrhage | 4 | 4 | 1.56% |
| Procedural pain | 7 | 5 | 1.95% |
| Rib fracture | 1 | 1 | 0.39% |
| Skin abrasion | 1 | 1 | 0.39% |
| Skin laceration | 7 | 6 | 2.33% |
| Suture related complication | 1 | 1 | 0.39% |
| Tooth injury | 2 | 2 | 0.78% |
| Upper limb fracture | 2 | 2 | 0.78% |
| Wound secretion | 1 | 1 | 0.39% |
| Investigations | **102** | **50** | **19.46%** |
| Alanine aminotransferase increased | 25 | 21 | 8.17% |
| Aspartate aminotransferase increased | 23 | 21 | 8.17% |
| Blood bilirubin increased | 2 | 2 | 0.78% |
| Blood cholesterol increased | 7 | 7 | 2.72% |
| Blood creatine phosphokinase increased | 1 | 1 | 0.39% |
| Blood creatinine increased | 1 | 1 | 0.39% |
| Blood glucose increased | 4 | 4 | 1.56% |
| Blood phosphorus decreased | 1 | 1 | 0.39% |
| Blood potassium decreased | 3 | 2 | 0.78% |
| Blood triglycerides increased | 1 | 1 | 0.39% |
| Blood urea increased | 3 | 3 | 1.17% |
| C-reactive protein increased | 11 | 11 | 4.28% |
| CSF white blood cell count increased | 1 | 1 | 0.39% |
| Gamma-glutamyltransferase increased | 1 | 1 | 0.39% |
| Glucose urine present | 1 | 1 | 0.39% |
| Heart rate increased | 4 | 4 | 1.56% |
| Hepatic enzyme increased | 1 | 1 | 0.39% |
| High density lipoprotein increased | 1 | 1 | 0.39% |
| Influenza A virus test positive | 1 | 1 | 0.39% |
| Low density lipoprotein increased | 1 | 1 | 0.39% |
| Oxygen saturation abnormal | 1 | 1 | 0.39% |
| Troponin I increased | 1 | 1 | 0.39% |
| Weight decreased | 4 | 4 | 1.56% |
| Weight increased | 1 | 1 | 0.39% |
| White blood cell count increased | 2 | 2 | 0.78% |
| Metabolism and nutrition disorders | **44** | **30** | **11.67%** |
| Decreased appetite | 10 | 10 | 3.89% |
| Diabetes mellitus | 3 | 3 | 1.17% |
| Diabetic ketoacidosis | 1 | 1 | 0.39% |
| Dyslipidaemia | 2 | 2 | 0.78% |
| Electrolyte imbalance | 1 | 1 | 0.39% |
| Fluid imbalance | 3 | 1 | 0.39% |
| Hyperlipidaemia | 2 | 2 | 0.78% |
| Hypokalaemia | 10 | 8 | 3.11% |
| Hyponatraemia | 8 | 7 | 2.72% |
| Hypophosphataemia | 3 | 2 | 0.78% |
| Shock hypoglycaemic | 1 | 1 | 0.39% |
| Musculoskeletal and connective tissue disorders | **330** | **148** | **57.59%** |
| Arthralgia | 50 | 43 | 16.73% |
| Back pain | 128 | 90 | 35.02% |
| Bursitis | 1 | 1 | 0.39% |
| Chest wall haematoma | 2 | 2 | 0.78% |
| Coccydynia | 12 | 11 | 4.28% |
| Dupuytren's contracture | 1 | 1 | 0.39% |
| Flank pain | 1 | 1 | 0.39% |
| Groin pain | 1 | 1 | 0.39% |
| Intervertebral disc protrusion | 1 | 1 | 0.39% |
| Limb discomfort | 2 | 2 | 0.78% |
| Muscle spasms | 2 | 2 | 0.78% |
| Muscle tightness | 1 | 1 | 0.39% |
| Muscular weakness | 4 | 3 | 1.17% |
| Musculoskeletal chest pain | 2 | 2 | 0.78% |
| Musculoskeletal pain | 21 | 18 | 7.00% |
| Myalgia | 9 | 9 | 3.50% |
| Neck pain | 15 | 14 | 5.45% |
| Osteoarthritis | 1 | 1 | 0.39% |
| Osteopenia | 2 | 2 | 0.78% |
| Osteoporosis | 1 | 1 | 0.39% |
| Pain in extremity | 72 | 54 | 21.01% |
| Periarthritis | 1 | 1 | 0.39% |
| Neoplasms benign, malignant and unspecified (incl cysts and polyps) | **5** | **4** | **1.56%** |
| Bladder neoplasm | 1 | 1 | 0.39% |
| Cholangiocarcinoma | 1 | 1 | 0.39% |
| Ovarian germ cell teratoma | 1 | 1 | 0.39% |
| Uterine leiomyoma | 2 | 2 | 0.78% |
| Nervous system disorders | **196** | **121** | **47.08%** |
| Altered state of consciousness | 1 | 1 | 0.39% |
| Arachnoiditis | 1 | 1 | 0.39% |
| brain hemorrhage | 1 | 1 | 0.39% |
| Brain injury | 1 | 1 | 0.39% |
| Carpal tunnel syndrome | 1 | 1 | 0.39% |
| Cerebral haemorrhage | 1 | 1 | 0.39% |
| Chorea | 1 | 1 | 0.39% |
| Dizziness | 27 | 23 | 8.95% |
| Haemorrhage intracranial | 1 | 1 | 0.39% |
| Headache | 131 | 93 | 36.19% |
| Hypoaesthesia | 11 | 8 | 3.11% |
| Loss of consciousness | 1 | 1 | 0.39% |
| Muscle contractions involuntary | 2 | 2 | 0.78% |
| Myoclonus | 1 | 1 | 0.39% |
| Neuralgia | 3 | 3 | 1.17% |
| Paraesthesia | 4 | 3 | 1.17% |
| Post herpetic neuralgia | 1 | 1 | 0.39% |
| Seizure | 3 | 3 | 1.17% |
| Sensory disturbance | 1 | 1 | 0.39% |
| Somnolence | 2 | 2 | 0.78% |
| Trigeminal neuralgia | 1 | 1 | 0.39% |
| Psychiatric disorders | **90** | **69** | **26.85%** |
| Affect lability | 5 | 4 | 1.56% |
| Anxiety | 14 | 14 | 5.45% |
| Completed suicide | 1 | 1 | 0.39% |
| Delirium | 2 | 2 | 0.78% |
| Depressed mood | 4 | 4 | 1.56% |
| Depression | 15 | 15 | 5.84% |
| Insomnia | 30 | 27 | 10.51% |
| Panic attack | 1 | 1 | 0.39% |
| Panic disorder | 1 | 1 | 0.39% |
| Sleep disorder | 17 | 16 | 6.23% |
| Renal and urinary disorders | **41** | **29** | **11.28%** |
| Acute kidney injury | 1 | 1 | 0.39% |
| Dysuria | 15 | 14 | 5.45% |
| Haematuria | 4 | 4 | 1.56% |
| Hydronephrosis | 2 | 2 | 0.78% |
| Micturition disorder | 4 | 4 | 1.56% |
| Micturition urgency | 1 | 1 | 0.39% |
| Nephrolithiasis | 4 | 3 | 1.17% |
| Nocturia | 2 | 2 | 0.78% |
| Pollakiuria | 4 | 4 | 1.56% |
| Proteinuria | 1 | 1 | 0.39% |
| Renal failure | 1 | 1 | 0.39% |
| Subcapsular renal haematoma | 1 | 1 | 0.39% |
| Urine abnormality | 1 | 1 | 0.39% |
| Reproductive system and breast disorders | **11** | **9** | **3.50%** |
| Acquired hydrocele | 1 | 1 | 0.39% |
| Benign prostatic hyperplasia | 2 | 2 | 0.78% |
| Pelvic pain | 7 | 6 | 2.33% |
| Scrotal oedema | 1 | 1 | 0.39% |
| Respiratory, thoracic and mediastinal disorders | **122** | **93** | **36.19%** |
| Asphyxia | 1 | 1 | 0.39% |
| Atelectasis | 1 | 1 | 0.39% |
| Chronic obstructive pulmonary disease | 1 | 1 | 0.39% |
| Cough | 23 | 19 | 7.39% |
| Dysphonia | 1 | 1 | 0.39% |
| Dyspnea | 2 | 1 | 0.39% |
| Dyspnoea | 13 | 13 | 5.06% |
| Haemoptysis | 2 | 2 | 0.78% |
| Hypercapnia | 1 | 1 | 0.39% |
| Nasal congestion | 2 | 2 | 0.78% |
| Nasal dryness | 1 | 1 | 0.39% |
| Oropharyngeal pain | 7 | 7 | 2.72% |
| Pharyngeal swelling | 1 | 1 | 0.39% |
| Pleural effusion | 2 | 2 | 0.78% |
| Pneumothorax | 2 | 2 | 0.78% |
| Productive cough | 22 | 20 | 7.78% |
| Pulmonary embolism | 2 | 2 | 0.78% |
| Reflux laryngitis | 1 | 1 | 0.39% |
| Respiratory arrest | 3 | 3 | 1.17% |
| Respiratory failure | 21 | 21 | 8.17% |
| Respiratory paralysis | 1 | 1 | 0.39% |
| Rhinalgia | 1 | 1 | 0.39% |
| Rhinitis allergic | 1 | 1 | 0.39% |
| Rhinitis hypertrophic | 1 | 1 | 0.39% |
| Rhinorrhoea | 7 | 7 | 2.72% |
| Septic pulmonary embolism | 2 | 2 | 0.78% |
| Skin and subcutaneous tissue disorders | **111** | **68** | **26.46%** |
| Acne | 1 | 1 | 0.39% |
| Blister | 3 | 3 | 1.17% |
| Decubitus ulcer | 3 | 3 | 1.17% |
| Dermal cyst | 4 | 2 | 0.78% |
| Dermatitis | 4 | 4 | 1.56% |
| Dermatitis contact | 9 | 9 | 3.50% |
| Dermatitis diaper | 1 | 1 | 0.39% |
| Dermatitis herpetiformis | 1 | 1 | 0.39% |
| Drug eruption | 1 | 1 | 0.39% |
| Dry skin | 1 | 1 | 0.39% |
| Eczema | 2 | 2 | 0.78% |
| Eczema asteatotic | 1 | 1 | 0.39% |
| Erythema | 7 | 7 | 2.72% |
| Hair colour changes | 1 | 1 | 0.39% |
| Ingrowing nail | 6 | 6 | 2.33% |
| Intertrigo | 1 | 1 | 0.39% |
| Neurodermatitis | 1 | 1 | 0.39% |
| Pemphigoid | 2 | 2 | 0.78% |
| Post inflammatory pigmentation change | 1 | 1 | 0.39% |
| Pruritus | 32 | 27 | 10.51% |
| Rash | 9 | 8 | 3.11% |
| Rash erythematous | 1 | 1 | 0.39% |
| Seborrhoeic dermatitis | 9 | 8 | 3.11 % |
| Skin exfoliation | 2 | 2 | 0.78% |
| Skin lesion | 4 | 3 | 1.17% |
| Skin weeping | 1 | 1 | 0.39% |
| Urticaria | 2 | 2 | 0.78% |
| Vitiligo | 1 | 1 | 0.39% |
| Surgical and medical procedures | **3** | **3** | **1.17%** |
| Gastrostomy tube removal | 1 | 1 | 0.39% |
| Tooth extraction | 2 | 2 | 0.78% |
| Vascular disorders | **24** | **20** | **7.78%** |
| Blood pressure inadequately controlled | 1 | 1 | 0.39% |
| Cyanosis | 3 | 3 | 1.17% |
| Hypertension | 3 | 3 | 1.17% |
| Hypotension | 12 | 10 | 3.89% |
| Phlebitis | 5 | 4 | 1.56% |
| Total | **1847** | **241** | **93.77%** |

Supplementary Table 3. Baseline characteristics of multiple-injection and single-cycle injection in the BM-MSC group

|  | Multiple-injection (n=21) | Single-cycle injection (n=134) | p-value |
| --- | --- | --- | --- |
| Age (yr) | 53.6±11.2 | 56.1±10.6 | 0.358 |
| Sex (M : F) | 13:8 (62%:38%) | 81:53 (60%:40%) | 1.000 |
| Riluzole used | 21 (100%) | 119 (88.8%) | 0.225 |
| Baseline ALSFRS-R score | 39.1±5.69 | 35.0±5.73 | 0.005 |
| Time from diagnosis to baseline (months) | 9.21±6.31 | 8.15±5.1 | 0.475 |
| Site of symptom onset (Bulbar:Limb) | 4:17 (19%:81%) | 29:105 (22%:78%) | 1.000 |
| Initial progression speed (pt/month) | 0.41 (0.25) | 0.68 (0.32) | <0.001 |
